# PySNV for complex intra-host variation detection

**DOI:** 10.1093/bioinformatics/btae116

**Published:** 2024-02-29

**Authors:** Liandong Li, Haoyi Fu, Wentai Ma, Mingkun Li

**Affiliations:** Key Laboratory of Genomic and Precision Medicine, Beijing Institute of Genomics, Chinese Academy of Sciences, and China National Center for Bioinformation, Beijing 100101, China; Key Laboratory of Genomic and Precision Medicine, Beijing Institute of Genomics, Chinese Academy of Sciences, and China National Center for Bioinformation, Beijing 100101, China; University of Chinese Academy of Sciences, Beijing 100101, China; Key Laboratory of Genomic and Precision Medicine, Beijing Institute of Genomics, Chinese Academy of Sciences, and China National Center for Bioinformation, Beijing 100101, China; University of Chinese Academy of Sciences, Beijing 100101, China; Key Laboratory of Genomic and Precision Medicine, Beijing Institute of Genomics, Chinese Academy of Sciences, and China National Center for Bioinformation, Beijing 100101, China; University of Chinese Academy of Sciences, Beijing 100101, China

## Abstract

**Motivation:**

Intra-host variants refer to genetic variations or mutations that occur within an individual host organism. These variants are typically studied in the context of viruses, bacteria, or other pathogens to understand the evolution of pathogens. Moreover, intra-host variants are also explored in the field of tumor biology and mitochondrial biology to characterize somatic mutations and inherited heteroplasmic mutations. Intra-host variants can involve long insertions, deletions, and combinations of different mutation types, which poses challenges in their identification. The performance of current methods in detecting of complex intra-host variants is unknown.

**Results:**

First, we simulated a dataset comprising 10 samples with 1869 intra-host variants involving various mutation patterns and benchmarked current variant detection software. The results indicated that though current software can detect most variants with F1-scores between 0.76 and 0.97, their performance in detecting long indels and low frequency variants was limited. Thus, we developed a new software, PySNV, for the detection of complex intra-host variations. On the simulated dataset, PySNV successfully detected 1863 variant cases (F1-score: 0.99) and exhibited the highest Pearson correlation coefficient (PCC: 0.99) to the ground truth in predicting variant frequencies. The results demonstrated that PySNV delivered promising performance even for long indels and low frequency variants, while maintaining computational speed comparable to other methods. Finally, we tested its performance on SARS-CoV-2 replicate sequencing data and found that it reported 21% more variants compared to LoFreq, the best-performing benchmarked software, while showing higher consistency (62% over 54%) within replicates. The discrepancies mostly exist in low-depth regions and low frequency variants.

**Availability and implementation:**

https://github.com/bnuLyndon/PySNV/.

## 1 Introduction

Virus proliferates rapidly in large numbers by replicating itself after infecting the host. During this process, due to the error-prone replication, the virus (particularly RNA virus) in the host develops a quasi-species state (Jary *et al.*, 2020) in which various mutated genomes coexist. The differential sites between these mutated genomes are called intra-host variations, including single nucleotide substitutions, insertions, and deletions. Additionally, similar phenomenon has also been noted in tumor tissues and mitochondria, where it is typically termed somatic mutation (Greenman *et al.*, 2007) or heteroplasmic mutation (Li *et al.*, 2010).

Within-host mutations have gradually received attention in viral studies, and studying the quasi-species phenomenon is helpful to understand the infection process and evolutionary characteristics of virus. When analyzing the severe acute respiratory syndrome coronavirus 2 (SARS-CoV-2) sequencing data, we observed that the intra-host variations of SARS-CoV-2 could be complex, including occurrences of long indels and adjacent or overlapped variants, which pose great challenges for accurately identifying these intra-host variants.

At present, the detection of intra-host variations mainly utilizes sequence alignment methods which identify the mutational composition of each site by comparing sequencing reads to the reference genome. However, sequencing alignment might not perform well in highly diverse regions (Zielezinski *et al.*, 2017). Alignment methods are usually designed for general tasks, and the parameter settings might not be optimal for complicated variant detection. For example, the detection of longer indels (insertion and deletion) from short-length reads of next-generation sequencing might be problematic unless the gap penalty is adjusted. Additionally, some adjoining or overlapping variants could combine into more complex variant sequences. These issues can increase the difficulties of detecting variants and estimation of frequencies within the host. Through experiments, we find that the current variant detection software is not fully capable of accurately detecting complex intra-sample mutations and their corresponding frequencies from sequencing samples.

Here, we present PySNV, an accurate variant caller with enhanced ability for detecting complex intra-host variants. PySNV is implemented in the Python programming language and utilizes several C libraries for computational efficiency. It accepts fasta or fastq sequencing files as input, and outputs the detected variant list (with position and variant bases), their estimated frequencies within the sample, and local depths at variant positions. Although currently PySNV does not support thread-level parallelism for a single sample, we provide a user-friendly data-level parallelism functionality, i.e. the user could input the directory containing the sample files, and PySNV would process the samples in parallel. The detailed methodology is described in the Section 2. We conduct experiments on both simulated and real datasets to evaluate the performance of PySNV, which are presented in the Section 3.

## 2 Materials and methods

### 2.1 Datasets

#### 2.1.1 SARS-CoV-2 sequencing data

The distribution of SARS-CoV-2 genome mutations at the population level was obtained by analyzing sequence variation data retrieved from the RCoV19 database (Li *et al.*, 2023) on 28 March 2023 (https://ngdc.cncb.ac.cn/ncov/?lang=en). We then collected and analyzed the SARS-CoV-2 targeted next-generation sequencing (tNGS) samples from the NCBI SRA database by 31 August 2021 (with other criterion described in Supplementary). We followed the HAVoC pipeline (Truong Nguyen *et al.*, 2021) while using VarScan2 (Koboldt *et al.*, 2012) as the variant caller, and identified a substantial number of complex intra-host variants. In total, 2367 and 2374 indels longer than 30 nt were detected, and 8% and 5% of indels had at least a neighboring variant within 10 nt range in the two types of data, respectively. These findings motivated us to explore the ability of current variant callers in detecting complex intra-host variants.

#### 2.1.2 Simulated dataset

**Figure 1.**
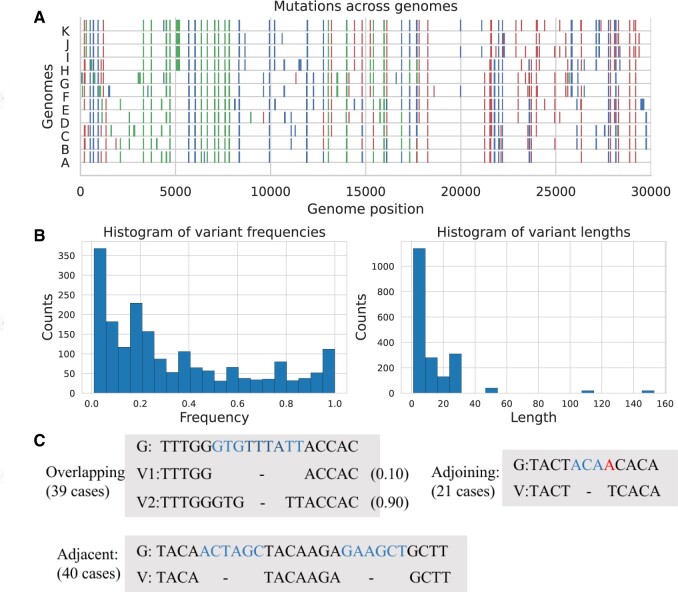
Description of simulated data. (A) Data generation: 11 manually generated SARS-CoV-2 genomes were pooled. We generated 10 test samples repeating this procedure with different set of frequencies. The colors represent different mutation types (Red: SNV, Blue: Deletion, Green: Insertion). (B) The distribution of ground truth frequencies and mutation lengths. (C) Examples of three types of neighboring variants that were included to increase detecting difficulties: adjoining (two or more variants are next to each other), adjacent (variants are close to each other), and overlapping (variant bases cover positions of other variants). G: reference genome sequence; V: variant sequences

To generate variants with a wide range of allele frequencies, we manually generated 11 haplotype genomes based on the reference genome (MN908947.3), so that their combinations could generate a wide range of variant frequencies between 0% and 100%. In addition to single substitution, deletion, and insertion mutations, their combinations were also included, which can be divided into three scenarios: overlapped (where different mutations happened at the same position); adjacent (where different mutations happened near each other within 20 nt); and adjoining (where different mutations happened next to each other). All variant scenarios were replicated at multiple frequencies and lengths with different mutations. We also incorporated several genome mutation hotspots identified from public-access sequence data, resulting in a total of 192 variant types across the SARS-CoV-2 genome (77 substitutions, 66 deletions, and 49 insertions), as illustrated in Fig. 1. We generated 10 samples using different frequencies (1%×1, 2%×2, 5%×3, 10%×2, 20%×3) assigned to the 11 haplotype genomes for testing, resulting in a total of 1869 variant cases with frequencies ranging from 2% to 100%. Among them, 618 indels had a length of 10 nt or more, and 355 indels were longer than 19 nt, 74 of which were longer than 29 nt. The maximum length of the designed indels was 153 nt, surpassing the length of the sequencing reads. The detailed information for all variants can be found in Supplementary Table S2.

Wgsim was used to generate simulation sequencing data from the 11 haplotype genomes, with read set as pair-ended 150 nt and an error rate of 1%. The generated reads were then mixed according to the frequencies of the 11 designed haplotype genomes. The simulated datasets have an average sequencing depth of 50 000× and were further downsampled to 5000×, 500×, 400×, 300×, 200×, 100×, and 50× for an ablation study. To avoid overfitting, we used different variants for method development and validation. For each variant, its frequency was calculated based on the summation of the abundance of reads carrying the variation.

#### 2.1.3 Replicate real data

We also randomly selected 99 pairs of SARS-CoV-2 surveillance tNGS replicate data from Tonkin-Hill *et al.* (2021) to evaluate the performance of the developed software (Supplementary Table S3).

### 2.2 Benchmarking

We benchmarked two reads alignment tools including MiniMap2 (Li 2018) and BWA-mem (hereinafter referred to as BWA) (Li 2013), with five variant callers: GATK-HaplotypeCaller (hereinafter referred to as GATK) (McKenna *et al.*, 2010), iVar (Grubaugh *et al.*, 2019), VarScan2 (Koboldt *et al.*, 2012), LoFreq (Wilm *et al.*, 2012), and FreeBayes (Garrison and Marth 2012) on the simulated data, and compared the accuracy of detecting variants (recall: true positives/(true positives + false negatives); precision: true positives/(true positives + false positives); and F1-score: 2 × (precision × recall)/(precision + recall)) and predicted frequencies. The detailed preprocessing tools and calling parameters could be found in Supplementary. Note that for GATK and FreeBayes, we need to set an appropriate ploidy value so that the callers can detect lower-level mutations and predict more precise frequencies. Further analysis was conducted on the 50 000× dataset to assess the factors affecting detection performance, by comparing the results for different variant lengths, frequencies and neighbor ranges. Then we analyzed the samples of different depths (5000×, and 500× to 50×) to evaluate the effect of sequencing depth on detection performance. For replicate real tNGS data, since ground truth was not available, we only analyzed the concordance between predictions made by the best existing callers and our proposed method.

### 2.3 Proposed method

**Figure 2.**
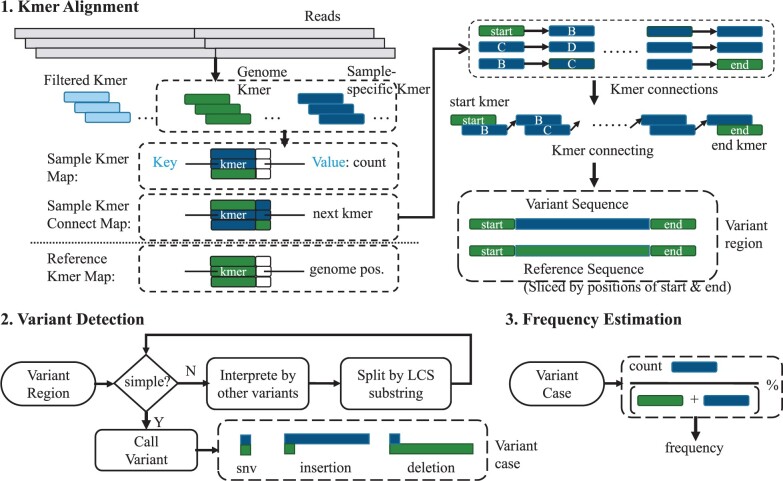
The overview of proposed method: (1) kmer alignment: utilizing Kmer Hashmaps to split sample reads kmers to genome kmers and sample-specific kmers, and connect sample-specific kmers to genome kmers to locate variant regions; (2) variant detection: firstly detecting simple variant in a variant region, then trying to interpret complex variant by simple variants or split complex variant to simple variants; and (3) frequency estimation: calculating the percentage of kmers which align the variant

The overall workflow of the proposed method is shown in Fig. 2. The method consists of four steps: kmer alignment, variant detection, frequency estimation, and quality assessment.

#### 2.3.1 kmer alignment

PySNV processes the reference genome and sample reads to build a reference kmer HashMap and a sample reads kmer HashMap. The sample kmers that derived from sample reads are split into two sets: genome kmers (kmers that can be found in the reference genome) and sample-specific kmers (kmers that cannot be found in the reference genome). In this study, we set the kmer length to be 21, a number that would not result in duplicate kmers in the SARS-CoV-2 genome (kmer sizes of 19 and 23 provided the same result).

Then, we traverse the reads again looking for three patterns of kmer connections: <genome_kmer, sample_specific_kmer>, <sample_specific_kmer, sample_specific_kmer>, and <sample_ specific_kmer, genome_kmer>. Based on these kmer connections, we could retrieve a connection of a series of kmers, which starts and ends with two genome kmers separately. We follow a depth-first search strategy to handle the situations when multiple kmers are connected to one kmer, as described in Algorithm 1 in Supplementary. The connection can easily be converted to a nucleotide sequence, which represents a variant region, whose first and last kmers are reference genome kmers that indicate the start and end positions of this region. Notably, sample-specific kmers with count lower than detection_threshold × local_depth (inferred using genome kmer count + sample_specific kmer count, at the position) are classified as low frequency kmers and ignored in the analysis (namely filtered kmer in Fig. 2). The occurrences of connected sequences are finally validated by string searching in the sample. The detection threshold in this study was set at 2%.

#### 2.3.2 Variant detection

For a pair of variant region sequence and its corresponding reference sequence identified in the previous process, the next task is to identify the variant type and exact altered bases. This is straightforward for a regular variant case which is the only variant in the region and is distant from its neighbor. Formally for a variant *V*, its type could be recognized by comparing the lengths of the reference and variant sequences:

(1) $$\mathrm{type}(V)=\{\begin{array}{cc} \prime \mathrm{deletion}\prime , & \mathrm{len}(\mathrm{ref}(V))>0,\mathrm{len}(\mathrm{var}(V))=0\\ \prime \mathrm{snv}\prime , & \mathrm{len}(\mathrm{ref}(V))=1,\mathrm{len}(\mathrm{var}(V))=1\\ \prime \mathrm{insertion}\prime , & \mathrm{len}(\mathrm{ref}(V))=0,\mathrm{len}(\mathrm{var}(V))>0\\ \prime \mathrm{complex}\prime , & \mathrm{otherwise} \end{array}$$

However, it could be complicated if the variants are in close proximity, e.g. adjoining, adjacent or overlapping. Considering these potential complex situations, we propose a three-step detection process: (i) Detect simple variant according to the criteria above for all the variant kmers detected in the region. (ii) Repeat step 1 after replacing the variant sequences by the reference bases resolved in step 1 (see Algorithm 2 in Supplementary material). (iii) For the remaining unresolved region variant sequences, find the longest common substring (LCS) between variant and reference sequence (minimum length: 5 nt); Then split the variant and reference sequences into two subsequences separately at the LCS. This step can be described by:

(2) $$\begin{array}{c} \{\begin{array}{c} \mathrm{ref}\_\mathrm{seq}\_1=\mathrm{ref}\_\mathrm{seq}[0:\mathrm{loc}\_\mathrm{ref}+L_{\mathrm{lcs}}]\\ \mathrm{var}\_\mathrm{seq}\_1=\mathrm{var}\_\mathrm{seq}[0:\mathrm{loc}\_\mathrm{var}+L_{\mathrm{lcs}}]\\ \mathrm{ref}\_\mathrm{seq}\_2=\mathrm{ref}\_\mathrm{seq}[\mathrm{loc}\_\mathrm{ref}:]\\ \mathrm{var}\_\mathrm{seq}\_2=\mathrm{var}\_\mathrm{seq}[\mathrm{loc}\_\mathrm{var}:] \end{array} \end{array}$$

where $L_{\mathrm{lcs}}$ is the length of longest common substring, and loc_ref and loc_var are the relative positions of *LCS* in the reference and variant sequences. For each pair of split variant and reference subsequence, PySNV would redo steps 1–3, as described in Algorithm 3 in Supplementary material. The cases that are not recognized in the above three steps could be tricky, as those remaining cases are either combinations of variants which usually have multiple explanations, or more complicated structural variants. We output these variant sequences and reference sequences directly instead of decomposing them into combinations of multiple variants.

#### 2.3.3 Frequency estimation

The next step is to calculate the frequency of each detected variant. Given kmers *K* mapped to the target genome position, let $K_{v}$ be the kmers matching the target variant case, $K_{o}$ be the kmers matching other variants also covering this position, $K_{g}$ be the kmers matching the reference genome, then the frequency of target variant case is

(3) $$\begin{array}{c} \mathrm{freq}(V)=\frac{\sum co\mathrm{unt}(K_{v})}{\sum co\mathrm{unt}(K_{v})+\sum co\mathrm{unt}(K_{o})+\sum co\mathrm{unt}(K_{g})} \end{array}$$

#### 2.3.4 Variant filtering

The final step is to filter out predictions likely to be caused by sequencing errors. Given local depth *n*, error rate ϵ, kmer count *x*, the binomial cumulative distribution function (CDF) is

(4) $$\begin{array}{c} \mathrm{CDF}(x)=\sum_{i=0}^{x}\left(\begin{array}{c} n\\ i \end{array}\right)\epsilon^{i}{\left(1-\epsilon \right)}^{n-i} \end{array}$$

For a variant *V*, given quality assessment threshold ξ, the criterion of passing the filter is

(5) $$\begin{array}{c} \mathrm{criterion}(V)=\mathrm{CDF}(\mathrm{count}(V))>\xi \end{array}$$

In this study, we set the threshold ξ to be 0.9999.

## 3 Results

### 3.1 Detection accuracy on simulated data

**Figure 3.**
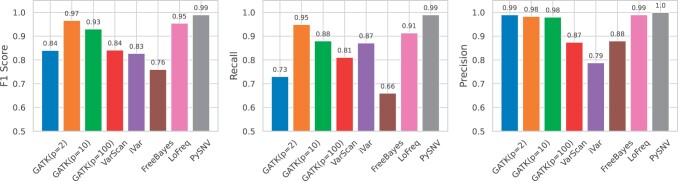
Benchmarking results of six intra-host variant callers on simulated data. Commands and parameters used to generate the data are provided in the Supplementary Methods. Variants with frequency lower than 2% were not considered in this analysis

The F1-score, recall, and precision of five existing callers, as well as our proposed software (PySNV) are shown in Fig. 3. For existing callers, the reads were aligned to the reference genome using Minimap2, which led to slightly better variant detection performance than BWA (Supplementary Table S1). GATK exhibited low sensitivity (recall 0.73) but high specificity (precision 0.99). The default value of ploidy might lead to ignorance of low frequency variants. Tuning of GATK’s ploidy parameter significantly enhanced sensitivity (up to 0.95 and 0.88 at *p* = 10 and *p* = 100, respectively), while retaining high specificity. Therefore, in the following experiments, we set *p* = 10 for GATK. VarScan demonstrated inferior sensitivity at 0.81 and specificity at 0.87, leading to a diminished F1 of 0.84. iVar achieved a sensitivity on par with GATK (*p* = 100) at 0.87, though at the expense of reduced specificity (0.79) owing to considerable false positive (FP) calls. Its F1 score of 0.83 was very affected by the FP predictions. FreeBayes exhibited moderate sensitivity and specificity of 0.66 and 0.88, resulting in an F1 score of 0.76. Despite that we also tuned several parameters including ploidy for FreeBayes, its performance was not comparable to other software. Thus we only reported one set of results of FreeBayes (see commands and parameters in the Supplementary Methods). LoFreq exhibited a sensitivity of 0.91 and a near-perfect specificity of 0.99, synthesizing a F1 metric of 0.95. Notably, LoFreq reported the greatest number of variants with frequencies lower than 2%, which were not considered in this assessment. Our proposed software, PySNV achieved the best sensitivity, specificity, and F1 exceeding 0.99 on the sample.

### 3.2 Frequency estimation correlation

**Figure 4.**
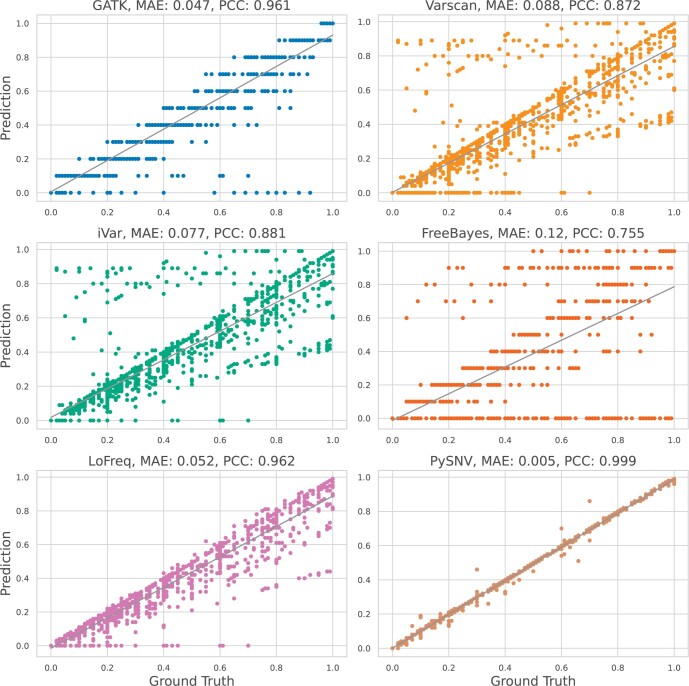
Prediction accuracy of the variant frequency by different callers. Mean absolute error (MAE) and Pearson correlation coefficients (PCC) were displayed above each figure

We then calculated the correlation between predicted frequencies and the ground-truth frequencies. The results are shown in Fig. 4. PySNV achieved the best correlation among all benchmarked callers, with a Pearson correlation coefficient of 0.999. This suggested a very accurate variant allele frequency (VAF) estimation. GATK also demonstrated good VAF correlation at 0.961 (ploidy number = 10), though it tended to be conservative and undercalling some ground truth variants. VarScan (0.872) and iVar (0.881) exhibited decent VAF correlation, but had more variability in accuracy across sites. LoFreq also displayed good VAF accuracy with a correlation of 0.962. In general, it matched ground truth frequencies across the spectrum, though tended to underestimate the frequencies. All tools but PySNV showed a drop in accuracy for lower frequency variants (<20%), likely reflecting challenges in sensitively detecting lower-level mutations.

### 3.3 Factors that affect the detection performance

**Figure 5.**
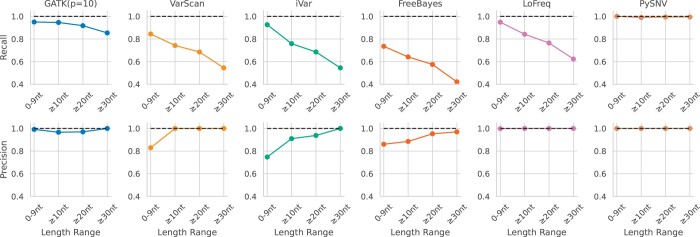
Performance of intra-host variant callers at different variant lengths

**Figure 6.**
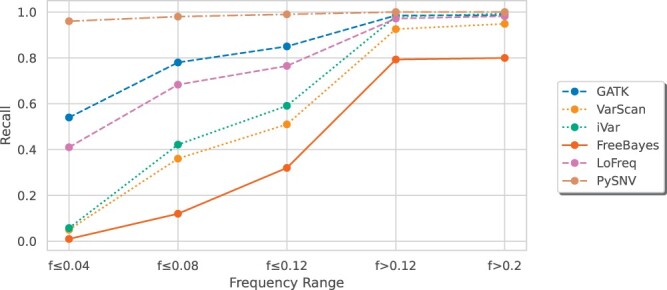
Performance of intra-host variant callers at different variant frequencies

**Figure 7.**
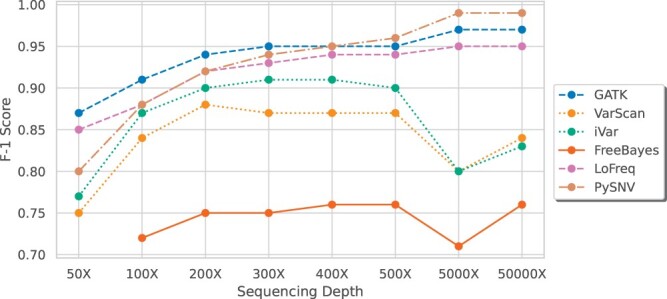
Performance of intra-host variant callers at different sequencing depths. F1-score of FreeBayes at 50× is below 0.5 and not shown on the figure

We conducted further analysis to discover the factors that might affect intra-host variation detection, including depth, variant length, neighbor variant distance, and frequency. These analyses were based on the previous test data except for depth analysis which used samples of different depths. The results in Figs 5–7 show that the recall of most callers dropped when the variant length increased or frequency decreased. Besides, most callers suffered from performance drops at very low depths. Meanwhile, neighbor variant distance did not show a clear pattern on affecting detection results (Supplementary Fig. S1).

For short variants (≤10nt), GATK, LoFreq and PySNV achieved high recall (>0.94) and near-perfect precision (>0.98). iVar had promising recall (0.93) but poorer precision (0.75). For longer variants (≥10nt), GATK and PySNV maintained perfect or near-perfect metrics across recall and precision, while LoFreq showed some decline in sensitivity. VarScan and iVar also suffered from declined sensitivity (around 0.75), though performed better with precision (1.0 and 0.9 separately). At ≥20nt, performance declined for VarScan, iVar, and FreeBayes, with recall dropping below 0.7. Meanwhile, PySNV preserved strong metrics while GATK showed a slight drop in recall. For very long variants (≥30nt), performance suffered for LoFreq, VarScan, iVar, and especially FreeBayes, with recall falling as low as 0.42. PySNV maintained high performance while GATK showed some decline in recall. In summary, PySNV achieved near-perfect recall of 0.99 and precision of 1.0, and GATK came closest with near-perfect metrics aside from a minor dip in long indel recall (Fig. 5).

Concerning the impact of frequency on variant detection, PySNV successfully detected more than 96% of variants across all frequency levels, whereas the performance of other methods decreased as the frequency decreased. At low frequencies (frequency ≤0.04), all tools except PySNV detected no more than 60% variants. VarScan, iVar, and FreeBayes suffered the most performance loss. At frequency ≤0.12, GATK and LoFreq achieved detection rates of 85% and 76%. Above 0.12, GATK, iVar, and LoFreq detected over 97% of variants, while VarScan and FreeBayes exhibited lower rates of 93% and 79%, respectively (Fig. 6).

In high sequencing depth (50 000×), PySNV achieved a near-perfect F1 score exceeding 0.99, while GATK and LoFreq also performed very well with F1 scores of 0.95–0.97. In contrast, other methods had F1 scores lower than 0.85. As the sequencing depth decreased, the performance of GATK, LoFreq, and PySNV declined, but was still better than iVar and VarScan (Fig. 7). Notably, PySNV, LoFreq, and GATK’s F1 scores all became lower than 0.95 when depth dropped to 200×. At very low depth (50×), PySNV suffered a performance drop with a F1 score of 0.87. LoFreq and GATK performed better with F1-scores of 0.91 and 0.93, respectably. This difference may be attributed to the increased vulnerability of kmer matching to errors compared to read alignment.

### 3.4 Computational resource

**Figure 8.**
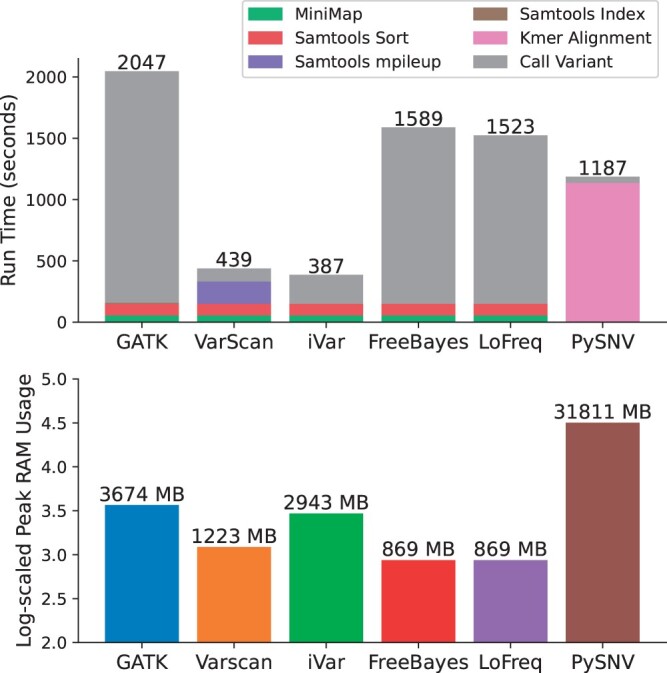
The running time and peak RAM usage of PySNV and benchmarked callers, on a workstation with dual Intel Xeon(R) 6248R CPUs

We conducted a comprehensive comparison of computational resources, including processing time and peak RAM usage, when processing a 50 000× sample with PySNV and other existing callers. All tools were executed in single-thread mode, and MiniMap2 was the alignment tool for all callers except PySNV. GATK was the slowest, taking 2047 s in total. VarScan and iVar were the fastest, completing in 439 and 387 s, respectively, followed by PySNV, LoFreq, and FreeBayes, which took 1187–1589 s (Fig. 8). The most time-consuming step for most callers is the variant calling step, while PySNV, being read-alignment-free, spent 1136 s on kmer alignment. Regarding RAM usage, PySNV exhibited the highest consumption due to the characteristics of kmer alignment, peaking at 31 811 MB memory. In contrast, read-alignment-based callers had a lower residence in RAM. Notably, LoFreq and FreeBayes consumed the least RAM, with peak usage occurring during the read sorting stage.

### 3.5 Comparison with GATK and LoFreq on real data

Since GATK and LoFreq showed good performance in the previous assessments, we compared PySNV against GATK and LoFreq using 99 actual replicate SARS-CoV-2 sequencing samples. GATK, LoFreq, and PySNV reported 27 185, 13 363, and 16 038 variants, respectively (with detection thresholds set at 2% for both LoFreq and PySNV). Among these, 5278 cases were concurrently reported by both GATK and PySNV, 8868 by both LoFreq and PySNV, 4942 by GATK and LoFreq, and 4300 by all three callers. The number of variants reported exclusively by LoFreq, GATK, and PySNV was 3854, 21 265, and 6192, respectively. Most of these uniquely reported variants had a relatively low frequency with 99%, 97%, and 99% of them exhibiting frequencies below 0.1, respectively, indicating difficulties in detecting lower-level mutations. More specifically, only one variant with a frequency above 0.1, consistently detected by both GATK and LoFreq in both replicates, was not reported by PySNV. This variant was identified at a depth of 5X.

**Figure 9.**
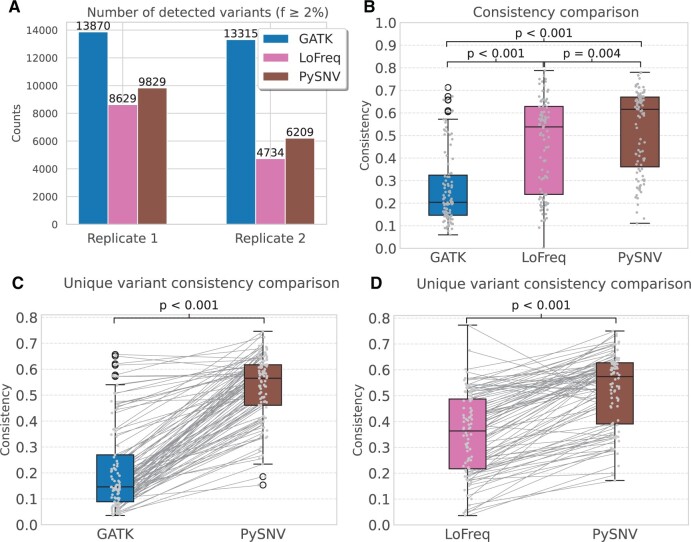
Comparison of the number and consistency of variants detected by GATK, LoFreq, and PySNV. (A) Comparison of the number of detected variants. (B) Comparison of consistency between replicate samples. (C) Comparison of the consistency of uniquely detected variants between GATK and PySNV. (D) Comparison of the consistency of uniquely detected variants between LoFreq and PySNV

We then analysed the consistency in variant detection across replicates for three callers, which serves as an indicator of the methods’ accuracy, since true mutations were consistently observed in both datasets. Notably, both LoFreq and PySNV exhibited a higher detection rate for variants in replicate1 samples compared to replicate2, while GATK detected a comparable number of variants in both replicates (Fig. 9A). GATK showed the least consistency between variants detected from replicates with a median of 20%, while PySNV demonstrated the highest consistency (median: 62%) (Fig. 9B). Furthermore, we also compared the consistency of uniquely detected variants, specifically comparing PySNV with GATK (Fig. 9C) and PySNV with LoFreq (Fig. 9D). The results revealed that the unique variants detected by PySNV exhibited a higher consistency compared to both LoFreq and GATK. Therefore, PySNV exhibited the highest accuracy, followed by LoFreq, and then GATK.

In addition, we assessed the impact of variant frequency, viral load (ct value) and sequencing depth on consistency between replicates for the PySNV and LoFreq methods (Supplementary Fig. S2). The analysis revealed a positive correlation between consistency and both variant frequency and sequencing depth, and a negative correlation with ct value, suggesting that better performance was achieved for high-frequency variants and samples with higher sequencing depths and viral loads. Meanwhile, we found the PySNV outperformed LoFreq in accuracy at different variant frequencies, sequencing depths and viral loads.

## 4 Discussion

### 4.1 Comparison of PySNV and other callers

Detecting intra-host variants is challenging due to the presence of low frequency variants, long indels, and complex combinations of different variants. Meanwhile, most variant callers were not designed for intra-host variants and lack evaluation of sensitivity and specificity for intra-host use. In this study, we simulated an intra-host variant dataset and benchmarked existing callers, including GATK, VarScan, iVar, FreeBayes, and LoFreq. The results showed that the existing callers were not fully capable of detecting the variants. Compared to our developed caller PySNV, the existing callers exhibited lower detection accuracy (F1-score) and frequency estimation accuracy (MAE and PCC). Our study also demonstrated that the callers’ detection abilities were limited for long indel variants and low frequency variants, except for PySNV.

PySNV’s advantages stem from the kmer alignment method and integrated design specifically tailored for intra-host variants. Most existing callers utilized third-party sequence alignment tools to map reads, thus being affected by alignment performance, which may be problematic in highly diverse regions (Zielezinski *et al.*, 2017). Callers cannot accurately detect variants if reads are incorrectly mapped initially. We employed a kmer alignment method to map reads, yielding promising results. Regarding run time, PySNV is comparable to current callers.

### 4.2 Limitations of PySNV

First, PySNV requires genome kmer to pinpoint the position of variants, a requirement that may pose challenges when sequencing depth is low, especially at the extremities of genomes. In addressing this issue, PySNV permits half-length matching of kmers on one side of the region, enhancing the likelihood of finding an anchoring genome kmer. Meanwhile, the absence of a high-quality reference genome could also lead to the unavailability of a genome kmer. Thus, we recommend assembling a consensus genome when the reference genome deviates significantly from the actual genome. In addition, the presence of replicate kmers in the reference genome obstructs the localization of the kmers, which may be resolved by exploring a kmer length that prevents such scenarios. Second, PySNV relies on a sufficient number of undisturbed kmers for accurate variant detection. However, clustered sequencing errors can introduce diversity among kmers in a region, resulting in a reduced count of kmers due to our filter for low-incidence kmers, which in turn weakens its detection ability. One potential solution is combining kmer-based alignment with read-based alignment to localize more kmers for variant detection. Third, the quality control method implemented in the pipeline is basic. Incorporation of additional variant filtering and quality assessment criteria may enhance performance. Fourth, PySNV allows variant detection within a sample, without using prior information about the distribution and types of intra-host and inter-host variants, which may aids in unraveling complex variants. Fifth, PySNV requires a significant amount of RAM to store and process kmers, preventing its use on computers and laptops with limited RAM.

### 4.3 Biological insights and implications of PySNV in variant analysis

PySNV exhibited the potential to boost our understanding and interpretation of viral evolution and adaption by providing superior performance in intra-host variants detection compared to existing software. Existing variant detection software, while effective for general mutations, struggled with complex intra-host variants like long indels and low-frequency mutations. PySNV tackles this challenge, demonstrating advantages by achieving near-perfect F1-score and highest correlation to ground truth frequencies on simulated data, and enhanced sensitivity and accuracy in real data by reporting more reproducible variants and achieving higher detection consistency. These advancements have critical implications for understanding viral dynamics including more precise evolutionary tracking, modeling transmission dynamic of low-frequency variants, and detecting complex mutations associated with drug resistance. Nevertheless, the performance of PySNV and other methods on real data is still limited, as indicated by the 62% consistency between replicates. This limitation might stem from PCR amplification bias, experimental stochasticity, and sequencing errors, underscoring the need for further efforts to improve the detection of intra-host variants.

We recognize the potential of extending PySNV to diverse types of data including other viruses, organisms, or data generated on different sequencing platforms. In theory, PySNV can be directly applied to various types of sequencing data if there are no replicates of kmers on the reference genome, as the presence of replicate kmers introduces ambiguity when connecting kmers and reconstructing the variant region. Notably, different types of sequencing data may exhibit variations in error types, error rates, and sequencing depths, potentially influencing detection accuracy in unexplored ways, which warrants further investigation.

## 5 Conclusion

PySNV performs well on both simulated and real data cases. It is capable of accurately detecting long indels and other complex intra-host variants, and demonstrates a remarkable ability to estimate variant frequencies.

## Supplementary Material

btae116_Supplementary_Data

## Data Availability

The Google Drive links to simulated sequencing samples that we used to evaluate the performance of PySNV are also available at the above link.

## Code availability

The Python source code of PySNV is available at https://github.com/bnuLyndon/PySNV/.
