# Supplementary material for: PySNV for complex intra-host variation detection
Source: Bioinformatics. 2024 Feb 29;40(3):btae116. doi: 10.1093/bioinformatics/btae116 (PMC10937218; doi:10.1093/bioinformatics/btae116)
Supplement: btae116_Supplementary_Data [file btae116_supplementary_data.zip › PySNV_supplementary_r2.pdf]

Supplementary of PySNV for complex intra-host variation detection

Liandong Li[1], Haoyi Fu[1,2], Wentai Ma[1,2], Mingkun Li[1,2*]

[1*]Key Laboratory of Genomic and Precision Medicine, Beijing Institute of Genomics, Chinese Academy of Sciences, and China National Center for Bioinformation, Beijing, 100101, China.
[2]University of Chinese Academy of Sciences, Beijing, 100101, China.

*Corresponding author(s). E-mail(s): limk@big.ac.cn;
Contributing authors: bnulee@hotmail.com; fhy11235813@gmail.com; mawentai2019m@big.ac.cn;

1 Supplementary Materials and methods

1.1 SARS-CoV-2 Sequencing Data

We analyzed variants in SARS-CoV-2 sequences downloaded from CNCB-NGDC RCoV19 database. 7407690 sequences were retrieved on 2023-Mar-28. Mutations that carried by at least three sequences were included for analysis, 71 insertions and 2296 deletions that have length over 30nt were detected (Figure S3).

We also analyzed SARS-CoV-2 raw sequencing data downloaded from NCBI SRA database on 2021-Aug-31. 151068 datasets were selected through the following criteria: pair-ended reads with lengths > 200; single-ended reads with lengths > 100; at least one variant (intra-host single nucleotide variant: iSNV) detected by VarScan2 readcounts (command: varscan readcounts sample.mpileup –min-coverage 0 –output-file sample.readcounts); data were generated on ILLUMINA platforms. Nine insertions and 2365 deletions that have length over 30nt were found in the samples (Figure S4).

1.2 Algorithms

Details of proposed algorithms are shown in Algorithm 1, 2 and 3.

1.3 Benchmarking of existing callers

We benchmarked five existing callers: GATK-HaplotypeCaller, iVar, VarScan2, LoFreq and FreeBayes on the simulated sample. The software dependencies and processing pipelines are listed in Figure S5. Note that for GATK and FreeBayes, we needed to set a proper value of ploidy so that the caller can detect intra-host variants and their frequencies.

The detail for each callers are:

GATK: The aligned sample reads were sorted and indexed by Samtools, and called by GATK (version 4.4.0) HaplotypeCaller. The numerical precision of GATK's predicted frequencies were affected by the ploidy parameter. For example, setting ploidy to 2 would only assign 0.5 and 1.0 as the variant frequency. Meanwhile, ploidy might also affect the detection accuracy. We tested 2, 10 and 100 as ploidy value to evaluate the best performance of GATK.

iVar: The aligned sample reads were sorted, indexed and piled up by Samtools, and called by iVar (version 1.4.2). iVar was designed for intra-host variant detection, with no ploidy parameter needed. The outputted variants with 'PASS = FALSE' were ignored for analysis.

VarScan: The aligned sample reads were sorted, indexed and piled up by Samtools, and called by VarScan (version 2.4.6) mpileup2cns. VarScan2 had been used for intra-host variant detection in some research, with no ploidy parameter needed.

FreeBayes: The aligned sample reads were sorted by Samtools, and called by FreeBayes (version 1.3.7). To act as a frequency-based pooled caller, we needed to set the ploidy and pooled-continuous parameter for FreeBayes. We tested 2, 10 and 100 as ploidy values. In this study, we only reported its best result using 10 as ploidy value.

LoFreq: The aligned sample reads were sorted by Samtools, and called by LoFreq (version 2.1.5). LoFreq was designed to be able to detect very low frequency variant, with no ploidy parameter needed.

1.4 Commands of tools and software

- Indexing reference genome using BWA:

  bwa index data/GCF_009858895.2_ASM985889v3_genomic.fna -p genome

- Alignment by BWA mem:

  bwa mem GCF_009858895.2_ASM985889v3_genomic.fna SampleP150_R1.fq SampleP150_R2.fq > mem-p150.sam

- Alignment by MiniMap2

  minimap2 -ax sr GCF_009858895.2_ASM985889v3_genomic.fna SampleP150_R1.fq SampleP150_R2.fq > aln_p150_mm.sam

- Sorting by Samtools:

  samtools sort mem-p150.sam > p150_sorted.sam

- Piling up by Samtools:

  samtools mpileup -aa -A -d 1000000 -B -Q 0 -f ref.fna p150.sam > p150.mpileup

- Call variant by GATK HaplotypeCaller:

  gatk HaplotypeCaller -R ref.fna -I p150_sorted.R.sam -O gatk_calls.vcf -ploidy 10

- Call variant by Varscan:

  varscan mpileup2cns p150.mpileup –min-var-freq 0.02 –variants 1 > varscan_calls.tsv

- Call variant by iVar:

  samtools mpileup -aa -A -d 1000000 -B -Q 0 p150_sorted.sam –reference ref.fna — ivar variants -p ivar_calls -q 20 -t 0.02

- Call variant by FreeBayes:

  freebayes -f ref.fna -p 10 -F 0.02 –pooled-continuous p150_sorted.sam > freebayes_calls.vcf

- Call variant by Lofreq:

  lofreq call –call-indels -f ref.fna -o lofreq_calls.vcf p150_lofreq_realign_indelq.bam

- Call variant by PySNV:

  python detect_sample.py –sample1 SampleP150_R1.fq –sample2 SampleP150_R2.fq –reference ref.fna –output output.csv –threshold 0.02

2 Supplementary Results

2.1 Detection consistency on replicate samples

We downloaded 99 pairs of replicate samples, and detected the variants using GATK, LoFreq and PySNV. For each pair of replicates, suppose c_1 is the detected variant counts of one caller on replicate1, and c_2 on replicate2. Let s be the count of variants that were detected in both replicates, then the consistency score of this caller on one replicate pair is calculated as $C = (s/c_1 + s/c_2)/2$. The scores across all replicate pairs were calculated and collected to measure the detection consistency of a caller.

Similarly, for unique variant analysis, supposing u_1 is the count of variants that only detected by caller A (comparing to caller B) on replicate1, and s_1 of u_1 are also detected on replicate2 (regardless of whether caller B has detected them on replicate2), then $C_1 = s_1/u_1$. The same procedure applies for calculating C_2 on replicate2, and the consistency score of unique detected variants of caller A over B is calculated as $C = (C_1 + C_2)/2$.

In addition, we evaluated the performance of LoFreq and PySNV on variants with different frequencies: ≥2%, ≥5% and ≥10%, and also on samples with different viral loads (ct values). To evaluate the influence of sequencing depths on real samples, replicate1 samples were classified into those with high sequencing depth (≥mean average depth) and those with low sequencing depth (<mean average depth). Their consistencys with replicate2 samples were then compared. The results are shown in Figure S2.

We also compared the processing time of GATK, LoFreq and PySNV on real data. Utilizing 4 kernels across all alignment tools and callers, the total processing time of the 99 replicate1 samples were 8.14, 51.9, and 3.51 hours for each caller, respectively. Notably LoFreq detected a substantial number of variants with frequencies below 0.01. GATK and LoFreq both used MiniMap2 for reads alignment, which took 3.07 hours, while PySNV spent 3.46 hours for kmer alignment.

2.2 Kmer alignment statistics

We collected the kmer alignment statistics of PySNV and MiniMap2 on both simulated and real datasets. In the case of the simulated pair-ended sample we examined, MiniMap2 successfully mapped 99.98% of the 10 million reads. Meanwhile, PySNV identified a total kmer count of approximately 1300 million, 76.85% kmers were mapped as genome kmer, 14.89% kmers were excluded due to low abundance, and the remaining 8.26% kmers were classified as sample-specific kmers. Notably, 99.85% of these sample-specific kmers were successfully connected as variant regions, and 99.95% of these regional kmers were ultimately reported as variant kmers (kmers belonging to variants over the detection threshold). The unused kmers were filterer out due to post-alignment frequency filtering.

On the 99 replicate1 samples where the average reads count was 2.17 million and the average kmer count was 463 million, MiniMap2 consistently mapped an average of 99.9% of reads. Meanwhile, PySNV reported an average genome kmer rate of 94.9%, an excluded kemr rate of 3.6%, and a sample-specific kmer rate of 1.5%. Notably,

99.2% of these filtered-in kmers were successfully connected as variant region, and 95.6% were reported as variant kmers.

Algorithm 1 DFS-based kmer connecting

Input:

- genome kmer HashMap M_g, kmer connection HashMap M_c, long kmer HashMap M_l
- a linked list of starting kmers K_s
- kmer length k, *reads_depth*, *detection_limit*

Output:

- starting ref position s_i, ending ref position e_i
- connected kmers K_c

$si \Leftarrow$ **query** $K_s[0]$ in M_g
initialize *Stack* ▷ *Stack*: a stack structure to store kmer lists
push K_s into *stack*
$C_{med} \Leftarrow \text{median}(\textbf{count}(K_s))$ ▷ C_{med}: median count
$\lambda \Leftarrow (reads\_depth[si] + C_{med}) * detection\_limit$
▷ λ: kmer count threshold
while *Stack* is not empty **do**
    $K_c \Leftarrow$ **pop** from *Stack* ▷ K_c: connected kmers
    $K_l \Leftarrow K_c[-1]$ ▷ K_l: lastly connected kmer
    if length$(K_c) > k$ **then**
        check_seq $\Leftarrow$ last k elements of K_c
        if *check_seq* not in M_l **then**
            continue to next iteration
        end if
    end if
    $ei \Leftarrow$ **query** k_l in M_g
    if ei is valid and $si \leq ei$ **then**
        output si, ei, K_c
    end if
    $K_n \Leftarrow$ **query** K_l in M_c
    ▷ K_n: the next kmers to connect
    if K_n is valid **then**
        for each K_i in K_n **do**
            if **count**$(K_i) \geq \lambda$ or K_i exists in M_g **then**
                $K'_c \Leftarrow K_c$ ▷ K'_c : new DFS branch
                insert K_i to K'_c
                push K'_c to *Stack*
            end if
        end for
    end if
end while

Algorithm 2 Interpret variant regions by detected variants

Input: variant set S, variant position loc, ref_seq and var_seq of variant region R
Output: interpreted var_seq

```
range[0] ⇐ loc                                              ▷ left endpoint
range[1] ⇐ range[0]+ length(ref_seq)                        ▷ right endpoint
for each V in S do
    snv_loc ⇐ position(V)                                   ▷ position of V
    snv_ref_len ⇐ length(var(V))                            ▷ length of var bases
    if range[0] ≤ snv_loc ≤ range[1] and snv_loc + snv_ref_len − 1 ≤ range[1] then
        rel_loc ⇐ snv_loc − range[0]
        var_seq2 ⇐ var(V)
        if var_seq[rel_loc : rel_loc + length(var_seq2)] = var_seq2 then      ▷ if R contains V
            ref_seq2 ⇐ ref(V)
            var_seq[rel_loc : rel_loc + length(var_seq2)] ⇐ ref_seq2          ▷ interpret R using V
        end if
    end if
end for
```

Algorithm 3 Variant detection

Input: variant regions queue Q
Output: variant HashMap M_v

```
initialize M_s
sort Q by mean kmer count                                   ▷ in descending order
while Q is not empty do
    R ⇐ pop from Q                                          ▷ R: a variant region
    if is_simple_region(R) then                             ▷ Eq.1
        V, K ⇐ detect_variant(R)                            ▷ Step 1 (Eq.1)
        for each (V_i, K_i) in (V, K) do
            if V_i not in M_v then:                         ▷ new variant case
                insert {V_i : [K_i]} to M_v
            else:                                           ▷ a new kmer for detected case
                L ⇐ query V_i in M_v                        ▷ query the kmer list of the variant
                insert K_i to L                             ▷ append new kmer to the list
                modify {Vi : L} to M_v
            end if
        end for
    else
        marker ⇐ interpret_by_detected_variants(R, M_v)     ▷ Step 2 (Algorithm 2)
        if marker = True then
            push R to Q
        else
            R_1, R_2 ⇐ split_region(R)                      ▷ Step 3 (Eq.2)
            push R_1, R_2 to Q
        end if
    end if
end while
```

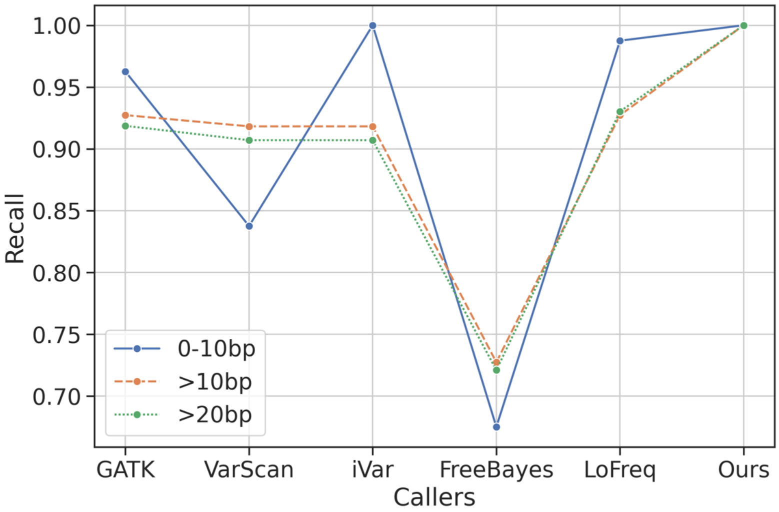

Fig. S1: Performance of intra-host variant callers at different neighbor distances.

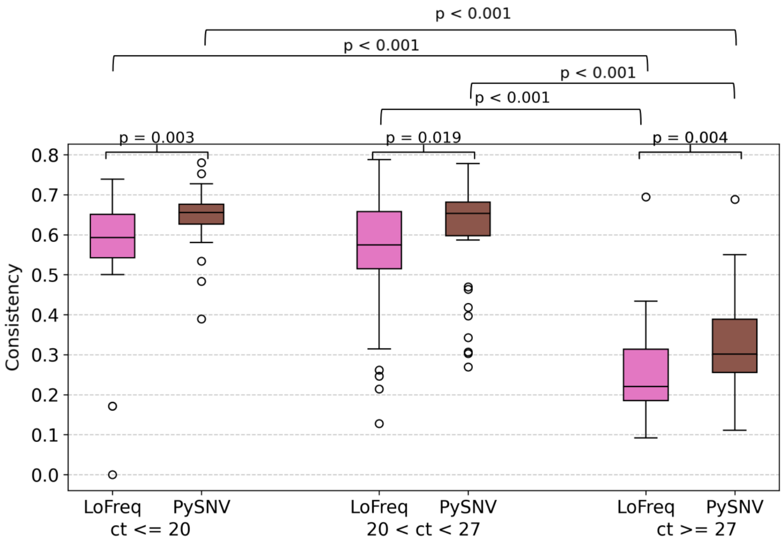

(a) Consistency at different ct values.

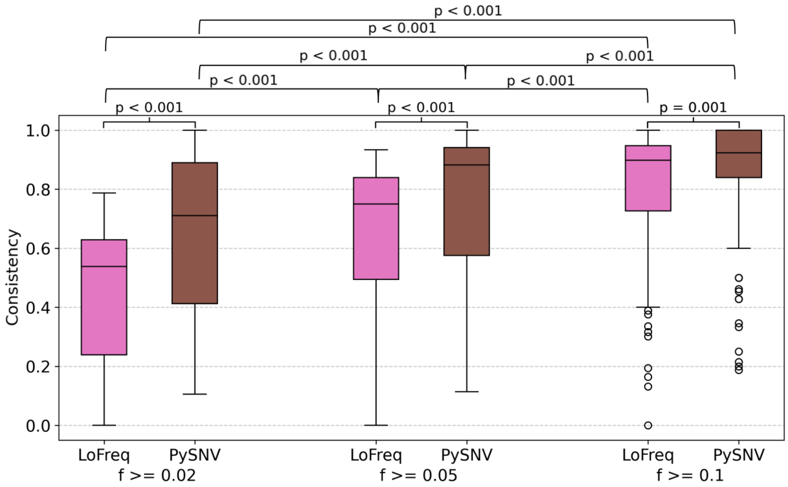

(b) Consistency at different frequencies.

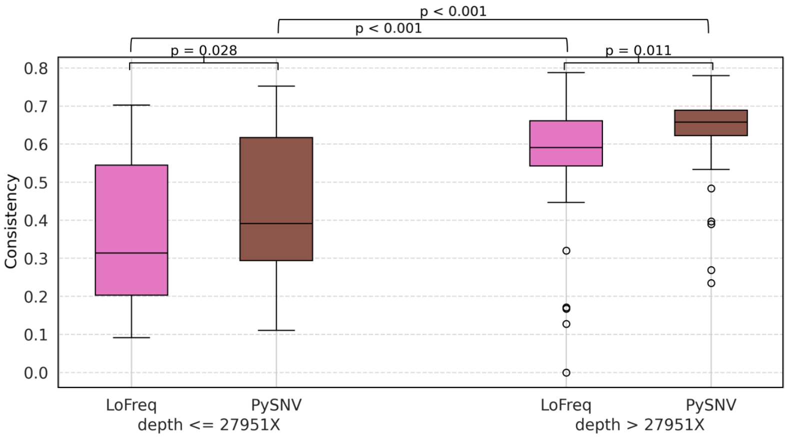

(c) Consistency of different depths of samples.

Fig. S2: Detection consistency between replicates at different ct values, frequencies and depths, compared between LoFreq and PySNV.

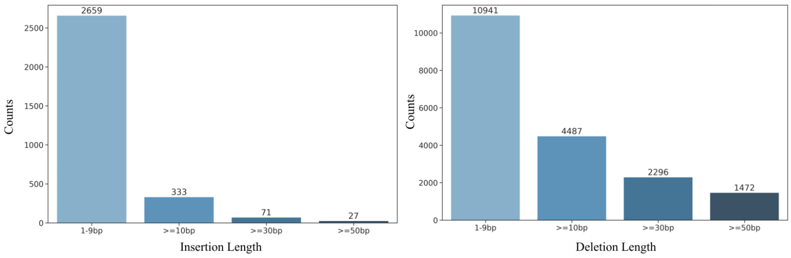

Fig. S3: Indel lengths of variants in sequence data.

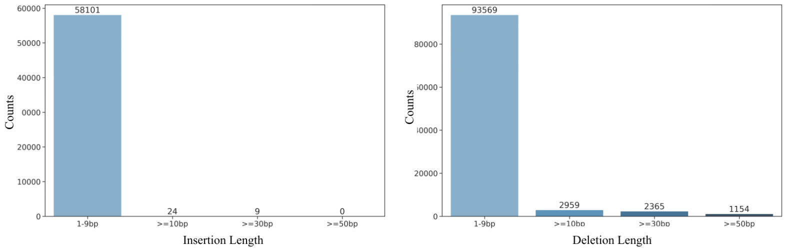

Fig. S4: Indel lengths of intra-host variants.

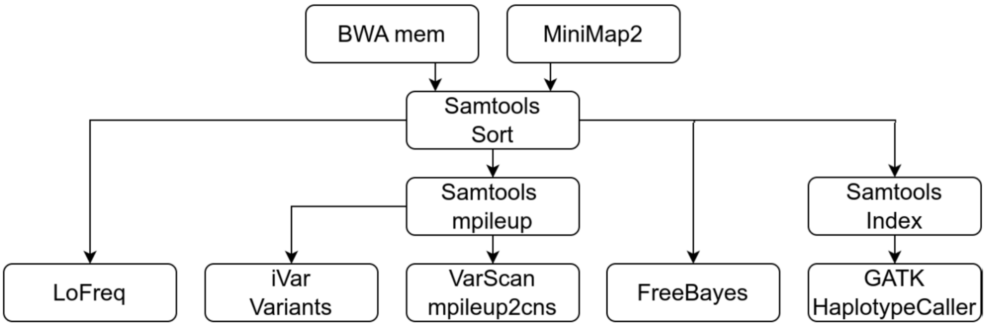

Fig. S5: Pipelines of benchmarked callers.
